# Experimental Study of Single-Lap, Hybrid Joints, Made of 3D Printed Polymer and Aluminium Adherends

**DOI:** 10.3390/ma14247705

**Published:** 2021-12-13

**Authors:** Przemysław Golewski, Marek Nowicki, Tomasz Sadowski, Daniel Pietras

**Affiliations:** Department of Solid Mechanics, Faculty of Civil Engineering and Architecture, Lublin University of Technology, Nadbystrzycka 38, 20-618 Lublin, Poland; p.golewski@pollub.pl (P.G.); t.sadowski@pollub.pl (T.S.); d.pietras@pollub.pl (D.P.)

**Keywords:** hybrid joints, single lap joints, 3D printing

## Abstract

This paper presents the results of an experimental study into single-lap joints. One part of the joint was made as a 3D printed polymer and had cylindrical tenons, while the other part was made of an aluminium flat bar having mortises whose diameter and distribution corresponded to the polymer tenons. In addition to the mechanical joint, a layer of double-sided VHB (Very High Bond) adhesive tape was also placed in the lap, thus creating a hybrid joint. In total, 80 specimens were made, which were divided into four groups: A—specimens with one tenon of different diameters, B—specimens with different number of tenons of the same diameter, C—specimens characterised by multi-stage operation and R—reference specimens, connected only by double-sided adhesive tape. The joints were subjected to uniaxial tensile tests. The force–displacement characteristics obtained and the energy required, up to the point of the failure of the joints, have been analysed in this paper. The four and six-stage joints designed can significantly increase the safety of the structures in which they will be used.

## 1. Introduction

The use of 3D printing is becoming increasingly popular, not only for the fabrication of mock-ups and prototypes, but also for industrial scale products. 3D printing technology facilitates production for multi-material products [1], reduces defects and lowers both production costs [2] and the need for repairs [3]. Considering the statistics, the Compound Annual Growth Rate (CAGR) for the next few years averages 23.5%. This means that the market will double every three years. Such significant growth also results in the greater involvement of researchers to improve this technology. However, not all parts can be replaced by printed products, since they may not be cost-effective, hence the problem of joining them with other materials arises. There are many joining techniques such as riveting [4], welding, spot-welding [5], friction stir welding (FSW) [6], friction stir interlocking (FSI) [7,8] and clinching [9]. The authors in [7] used micro-texturing by ablative laser pre-treatment on the surface of an aluminium alloy. In this case, the polymer–aluminium bonding was done using the FSW method, which produced high strength. In the paper [10], the authors used the Injection Clinching Joining (ICJ) method in which a pre-formed polymeric tenon is melted and formed in the mortise of a metal part. This type of mechanical joint is free of clearance. A similar technique is presented in [11], but in this case an aluminium tenon, made by a printing technique on the surface of a thin-walled profile, is used. At present, the laser-assisted technique is also used for joining metal-printed polymer structures [12,13]. During this process, the polymer is melted under the influence of laser beam energy and penetrates the structure of the metal surface under the application of a specific joining pressure. The advantage of this type of solution is that there is no correlation between the surface roughness Ra and the shear strength of the joint. A similar technique is ultrasonic welding [14], which allows thermoplastic composites to be joined with aluminium alloys. In the work [15], the authors used a clinched metal-polymer type joint. Experimental studies were conducted by varying the main process parameters such as heating time, the temperature of the air and moulding pressure.

A common feature of these types of mechanical connections is that they invade the structure of the parts being joined. In many cases, they are also a source of concentrated stress. Therefore, another group of joints that can be used to connect polymer 3D prints are adhesive joints, either using liquid adhesives [16,17], tapes [18] or dual-adhesive [19,20]. Their advantage is that the load is transferred over a considerable area and thus distribution of the stress, in the joined parts, is in many cases free of concentration.

Currently, the study of the adhesive bonding of polymer 3D prints has been carried out in many directions. The works [21,22] analysed the effect of surface topography on peel and shear strength. The surface topography can also be designed, for example, by introducing undulation [23]. When using adhesive joints, the thickness of the adhesive plays a large role. In the paper [24], three adhesive thicknesses were analysed: 0.2 mm, 0.3 mm and 0.4 mm. It was shown that the 0.2 mm thickness was the best and led to higher mechanical performance and tensile strength compared to the other two. The bonding strength also depends on the adhesive used. When bonding polymer 3D prints, it is more beneficial to use cyanoacrylate adhesive than epoxy adhesive [25]. Surface wettability, which is another parameter, can be modified by sandblasting or by employing atmospheric plasma treatment; using the second, an increase of 200% in shear strength can be achieved [26].

In recent years, connections using double-sided adhesive tapes have received increasing attention [27,28,29,30,31]. In many cases, they allow traditional liquid adhesives or mechanical joints to be replaced. From a researcher’s point of view, the soft and relatively thick (0.4–1.6 mm) layer of double-sided adhesive tape also comes with some challenges. The acrylic-based polymer used in VHB tapes is highly deformable, highly visco-elastic and very sensitive to temperature changes, hence the numerical modelling of this type of bond is difficult. 

The simultaneous use of two techniques: mechanical and adhesive joints lead to a hybrid joint [32,33]. In this case, research is usually conducted using a single-lap scheme and various types of mechanical fasteners such as screws [34,35,36], embossing [37] and spot-welding [38].

To sum up, many techniques are used to join polymer 3D prints. Authors most often aim to increase their strength and often the energy used, up to the point of the failure of the joint, is also analysed. However, there is not much call to use the two simplest solutions: double-sided adhesive tape and mechanical connection of the “mortise and tenon” type. This type of hybrid solution is the subject of this paper. Its advantage is the rapidity with which the joints can be produced, since no adhesive curing time is needed and also, by properly selecting the ratio of the surface of the mechanical joint to the surface of the double-sided adhesive tape, it is possible to design the characteristics of the joint. In this paper, the results of the laboratory tests for innovative joints, with multi-stage characteristics, are also presented.

## 2. Materials and Methodology

**Figure 1:**
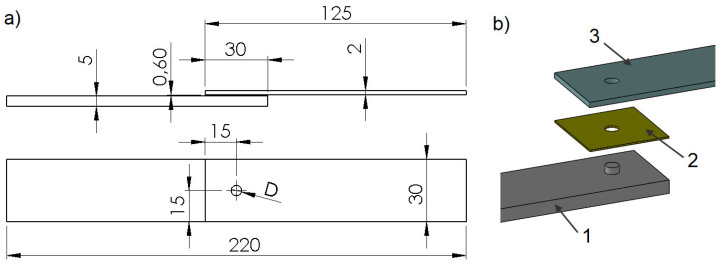
Joints with one tenon: (**a**) joint diagram; (**b**) perspective view (1—polymer lap, 2—double-sided adhesive tape, 3—aluminium lap).

**Figure 2:**
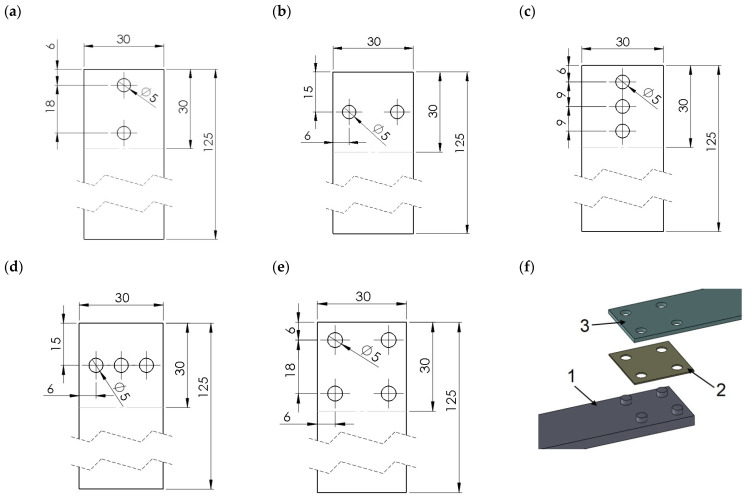
Types of joints with different numbers of tenons of the same diameter D = 5 mm: (**a**) model B1; (**b**) model B2; (**c**) model B3; (**d**) model B4; (**e**) model B5; (**f**) model B5 in perspective view (1—polymer lap, 2—double-sided adhesive tape, 3—aluminium lap).

**Figure 3:**
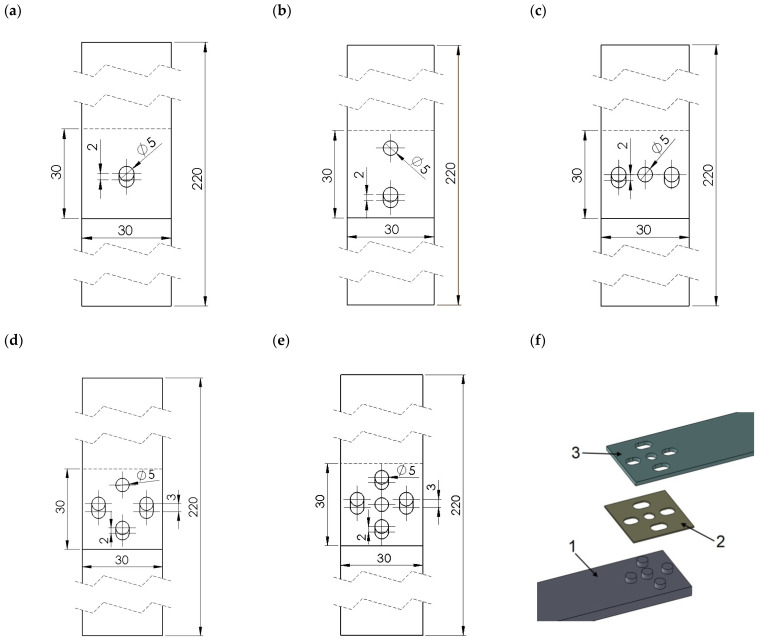
Special joints characterised by multi-stage operation using tenons of diameter D = 5 mm: (**a**) model C1; (**b**) model C2; (**c**) model C3; (**d**) model C4; (**e**) model C5; (**f**) model C5 in perspective view (1—polymer lap, 2—double-sided adhesive tape, 3—aluminium lap).

Testing of the hybrid joints was performed using the popular and currently most widely used single-lap model. The dimensions of the joints, which are shown in Figure 1, Figure 2 and Figure 3, were designed so that there would be no fracturing of the connected parts. 

The hybrid joints were divided into 3 groups:

- group A—single-lap joints with one tenon having 5 different diameters “D”: 5 mm, 8 mm, 11 mm, 14 mm and 19 mm (Figure 1),
- group B—joints with different numbers of tenons of the same diameter D = 5 mm (Figure 2),
- group C—special joints, characterised by multi-stage operations with tenons of diameter D = 5 mm (Figure 3).

In addition, reference joints “R” were made, using only double-sided adhesive tape, having the same dimensions as in Figure 1. In each case, 5 specimens were used per batch, hence the total number of specimens was 80. 

3D prints of polymer laps, made of Z-ABS material including the tenons, were made on a Zortrax M300 printer. The print parameters and material data of the filament are shown in Table 1.

Although the tensile strength of the ABS material according to Table 1 is 29.6 MPa, tests conducted on dog bone samples with a cross section of 10 × 5 mm, showed a print tensile strength of 13.25 MPa.

The second laps were made of an aluminium flat bar, made of AW-6060 T4 alloy, with the following properties: yield strength—65 MPa, tensile strength 130 MPa, elongation at break 13%. The holes in the aluminium laps were made by milling on a CNC machine. 

To connect the aluminium and polymer parts, 3 M VHB 5925 double-sided adhesive tape with a thickness of 0.6 mm and a width of 38 mm was used. The tape is designed for permanent bonding to irregular surfaces or to powder-skin coated materials and provides good adhesion, with high and medium surface energy to various surfaces, including plastics, paints, metals and glass. One of the mechanisms of adhesion of VHB tape to laps is intermolecular force. This is an electrical interaction that occurs when two molecules are in close proximity to each other. The two surfaces do not stick to each other regardless of how close they are. The reason for this is that the surfaces are rough at the nano level and the molecules cannot get close to each other. The VHB tape causes these roughnesses to fill in, resulting in intermolecular forces. 

The manufacturing process of the joints consists of the following steps:

1. A check was done to make sure that all mechanical joints would be able to fit together. If there was significant compression, the geometry had to be corrected.
2. The surface of the aluminium laps was cleaned with Loctite 7061 because coolant and oil were to be used during milling.
3. UNI UV 0.23 primer for VHB tapes was applied to the surfaces of the aluminium and polymer laps.
4. The tape was unrolled and placed on a flat surface with the adhesive side up and then five aluminium laps were placed on it, next to each other, at intervals of a few millimetres.
5. Cutting the tape for each lap was done using a scalpel and a punch of appropriate diameter; holes were made in the tape to match the corresponding holes in the aluminium lap.
6. The protective layer was removed from the tape and bonded to the other polymer lap.
7. The tape was 38 mm wide and the overlay was 30 mm wide. The excess tape on the aluminium part was trimmed with a scalpel and then removed from the surface. In this way, the tape’s geometry perfectly matched the geometry of the lap.
8. The tape manufacturer requires a low clamping pressure of 100 kPa, hence the laps were clamped by hand without any additional tooling. The specimens were tested more than 72 h after they were made, which, according to the manufacturer, ensures that maximum strength is achieved.

**Figure A1:**
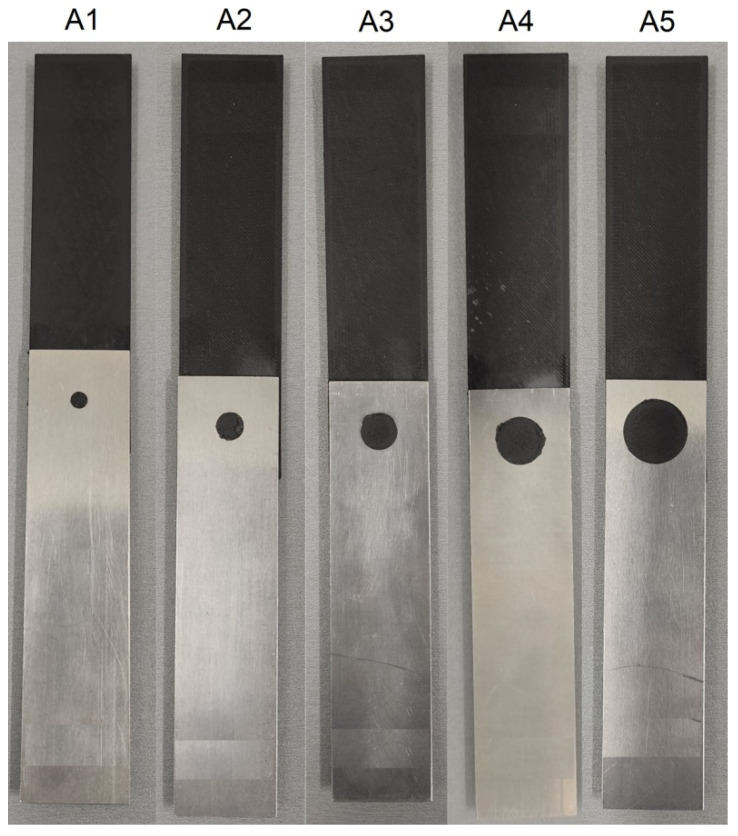
Samples A before the test.

**Figure A2:**
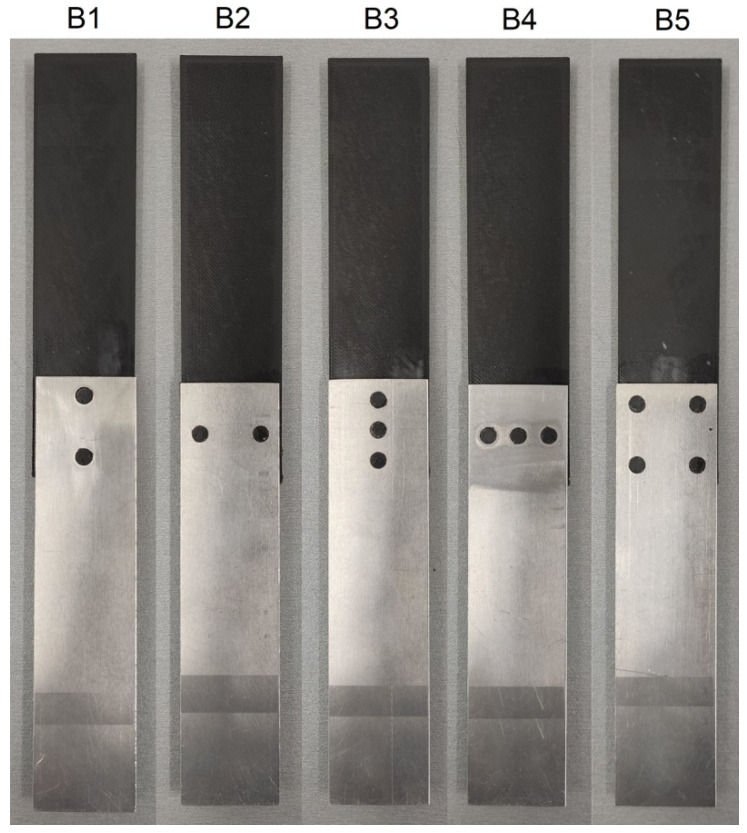
Samples B before the test.

**Figure A3:**
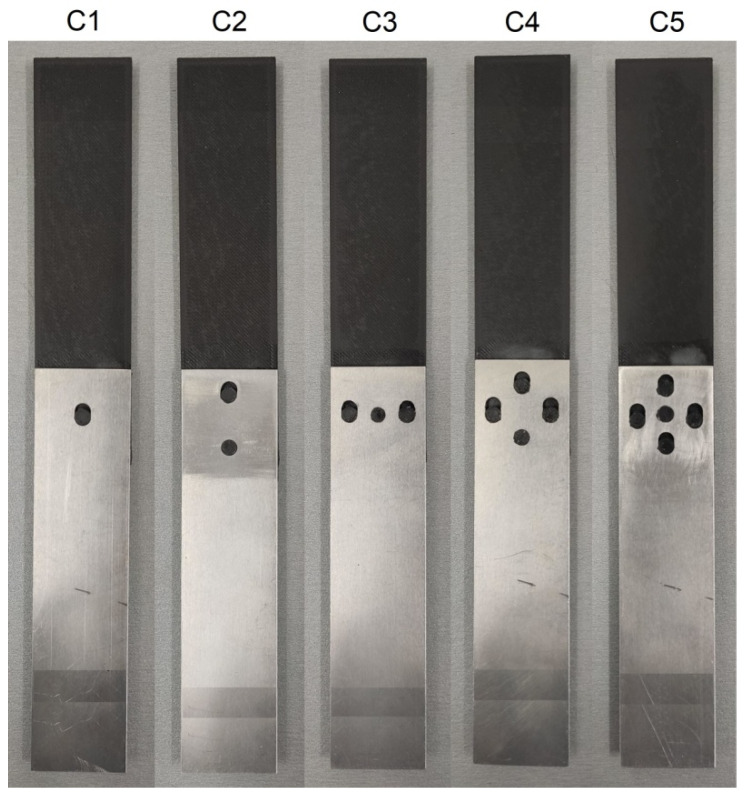
Samples C before the test.

For this type of connection, the strength of the laps exceeded the strength of the connection, hence there was no risk of breakage nor was there any need for bonding the tabs at the ends of the laps. The completed connections (one specimen from each batch) are shown in Figure A1, Figure A2 and Figure A3. 

Uniaxial tensile tests were performed on an MTS 25 kN testing machine. In order to avoid pre-bending, the specimens due to the geometry of the single-lap model, a sufficient thickness of inserts was used between the grips and the ends of the tabs. The specimens were loaded at a constant displacement increment of 5 mm/min. Tests were conducted at a temperature of 23 °C ± 2.

## 3. Results and Discussion

### 3.1. Mechanical Response of Reference Specimens “R” Joined by VHB Double-Sided Adhesive Tape

**Figure 4:**
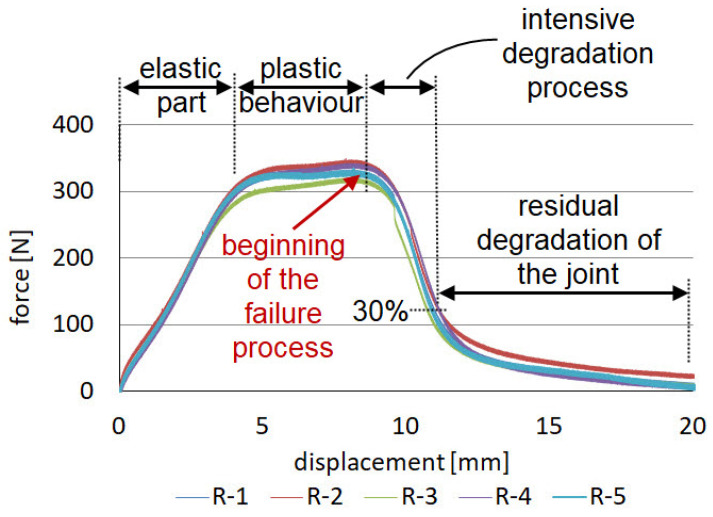
Force–displacement diagrams for reference specimens “R” joined by VHB double-sided adhesive tape.

**Figure A4:**
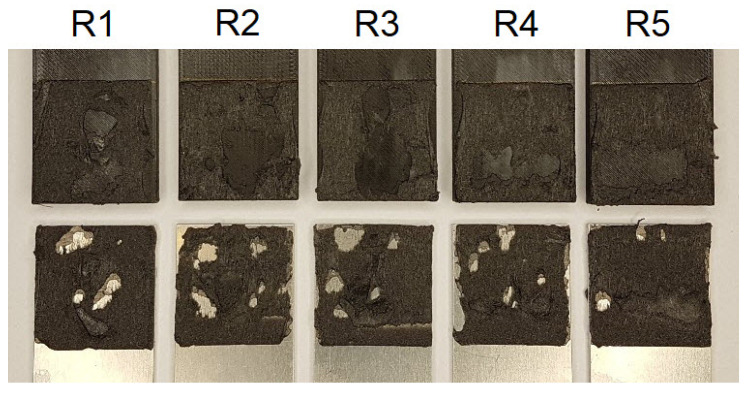
Reference samples after the tensile test.

Although VHB double-sided adhesive tapes are characterised by low maximum stress, high strains to failure result in impressive fracture energy. The failure process often starts with the nucleation of microscopic cracks inside the layer. The failure model is usually cohesive, with a layer of tape remaining on both adherends. Operation of the adhesive tape, as shown in Figure 4 for the reference specimens, has several stages. Initially, the force increases linearly; the graph then becomes curved and flattened. This stage continues until about the middle of the strain range, culminating in failure. The final stage is the tape failure process, which initially occurs quite rapidly; however, at about 30% of the maximum force, the graph begins to smoothly move towards zero. The average maximum force, achieved for the joints made with the VHB tape, was 333 N, which corresponds to a stress of 2.22 MPa in the 3D printed laps. The average energy required to failure, calculated as the area under the force–displacement diagram, was 3 J. These values will be considered as reference and the average values of force and energy for each batch of specimens and work stage will be referred to. The samples after the test are shown in Figure A4. The failure is mainly cohesive in nature. There is also a loss of adhesion between the tape and the aluminum surface and between the tape and the polymer 3D print. The percentages of adhesion failure are 8% and 17.4%, respectively, considering all samples in the batch. 

### 3.2. Mechanical Response of the Single—Lap Joints with One Tenon—Group “A”

**Figure 5:**
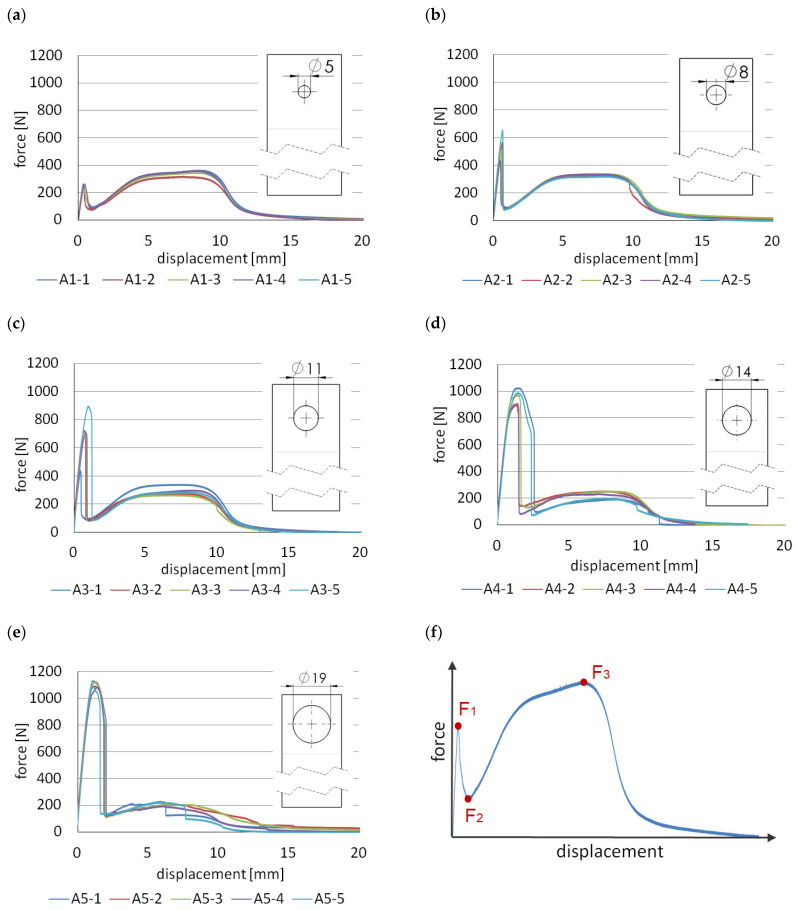
Force–displacement diagrams for models “A”: (**a**) models A1; (**b**) models A2; (**c**) models A3; (**d**) models A4; (**e**) models A5; (**f**) working diagram.

The first type of hybrid joint, labelled “A”, was concerned with analysing the effect of the diameter of the mechanical joint. In this case, only one tenon was used, whose diameter varied from 5 mm to 19 mm. In batches A1 to A4, there was shearing of the mechanical joint and subsequent gradual degradation up to the final failure of the adhesive joint. In the case of specimen A5, the mechanical joint was so strong that the lap breakage occurred at the location of the tenon. By properly selecting the ratio of the area of the tenon and the adhesive tape, not only can different levels of maximum force and energy be obtained, but different characteristics can also be obtained. An analogous phenomenon occurs here, as in [19], where double-sided adhesive tape was also used, while the role of rigid connection was achieved by the point bond of epoxy adhesive. Thus, the “A” type joints are characterised by a two-stage operation (Figure 5), which is schematically shown in Figure 5f. In the first stage, the rigid mechanical joint works and the load are mainly transferred through the polymeric tenon reaching the F_1_ force. When the tenon is cut by shear loading, the force drops rapidly to F_2_, while the joint does not lose integrity. The load is taken up by the adhesive joint and the force increases, reaching F_3_ again.

The purpose of using polymeric tenons is to increase the strength and stiffness of the joint. For the A1 group of specimens, the maximum force achieved by the mechanical connection is about 26% lower than the maximum force achieved by reference connection “R”.

When the diameter is increased to 8 mm, the mechanical joint achieves a higher strength, by 46.5%, compared to the reference connection. For A3 series specimens, having a tenon diameter of 11 mm, the mechanical joint achieves about 123% of the maximum strength of the reference connection “R”. 

Although for the subsequent A4 group, this ratio is even more favourable and the connection has a higher strength, it should be noted that the strength decreases quite sharply in the working stage of the adhesive joint. For the A1 and A2 series, the work of the adhesive tape is similar to that of reference joint “R”. There is no noticeable decrease in strength, in this case. The reason for this is that the area occupied by the tenon is small (11%) relative to the area of the entire lap. For A4 and A5, the adhesive area of the tape decreases by about 17.1% and 31.5%, respectively.

Table 2 collects the force values F_1_–F_3_ and the corresponding stress values σ_1_–σ_3_ in the 3D printed lap outside the joint zone, assuming that it is only in tension.

**Figure A5:**
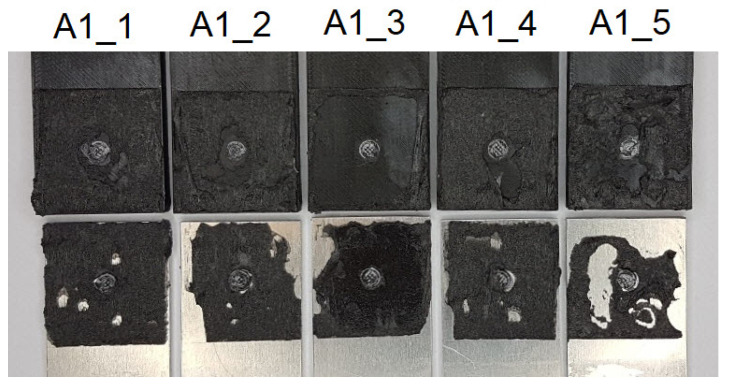
Samples A1 after the tensile test.

**Figure A6:**
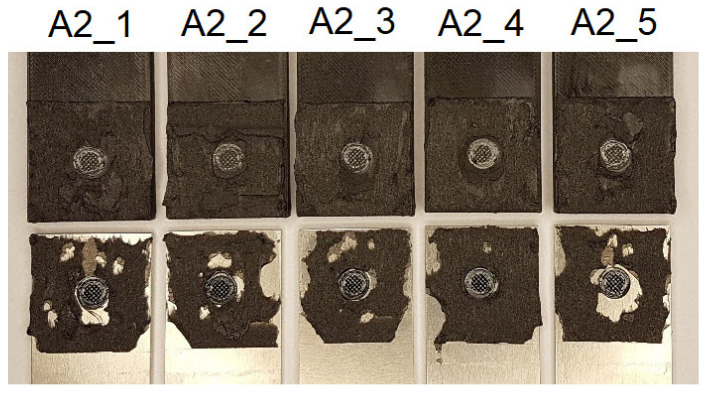
Samples A2 after the tensile test.

**Figure A7:**
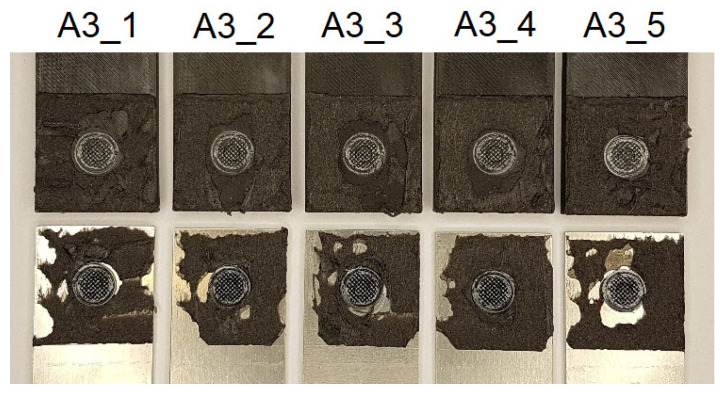
Samples A3 after the tensile test.

**Figure A8:**
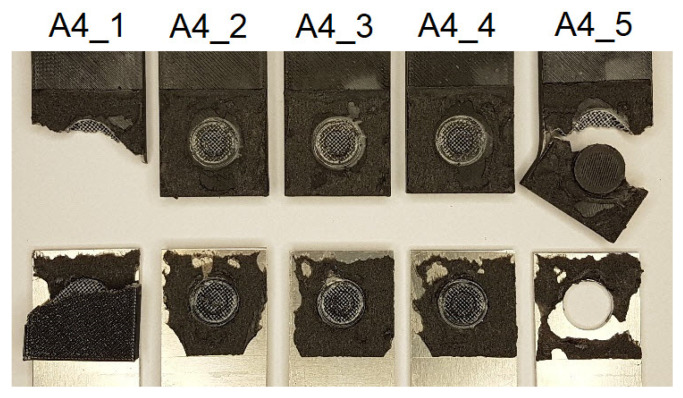
Samples A4 after the tensile test.

**Figure A9:**
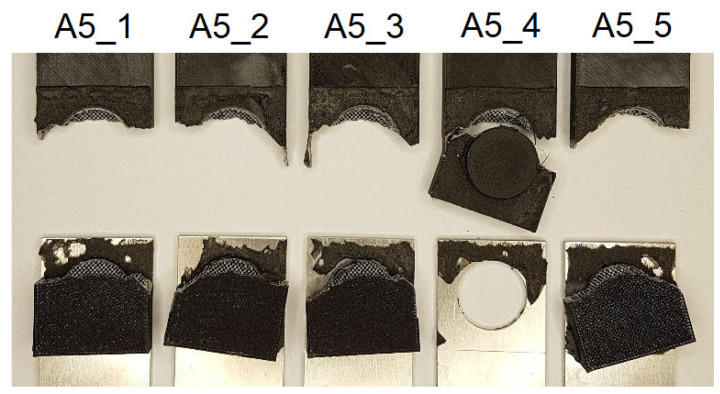
Samples A5 after the tensile test.

Group A2 should be considered as the best model because, in this case, we obtain a significant increase in stiffness in the initial stage of operation, which does not come at the expense of a decrease in the strength of the adhesive joint. Considering the energy which is needed to the point of the failure of the joint, the A2 model is also, in this respect, the best. An increase of about 6.6% is obtained compared to the reference model. In general, the highest energy was obtained for model A5, but it should be noted that, in this case, there was no shear failure of the mechanical connections, but rather a fracture of the lap at the tenon location. The samples after the test are shown in Figure A5, Figure A6, Figure A7, Figure A8 and Figure A9. As in the reference samples, there is mainly cohesive failure in the tape. There is also a loss of adhesion between the tape and the aluminium surface and between the tape and the polymer 3D print. For example, for samples A1 (Figure A5), the percentage of adhesive failure is 16% and 15%, respectively, considering all samples in the batch.

### 3.3. Mechanical Response of the Joints Having Different Numbers of Tenons—Group B

**Figure 6:**
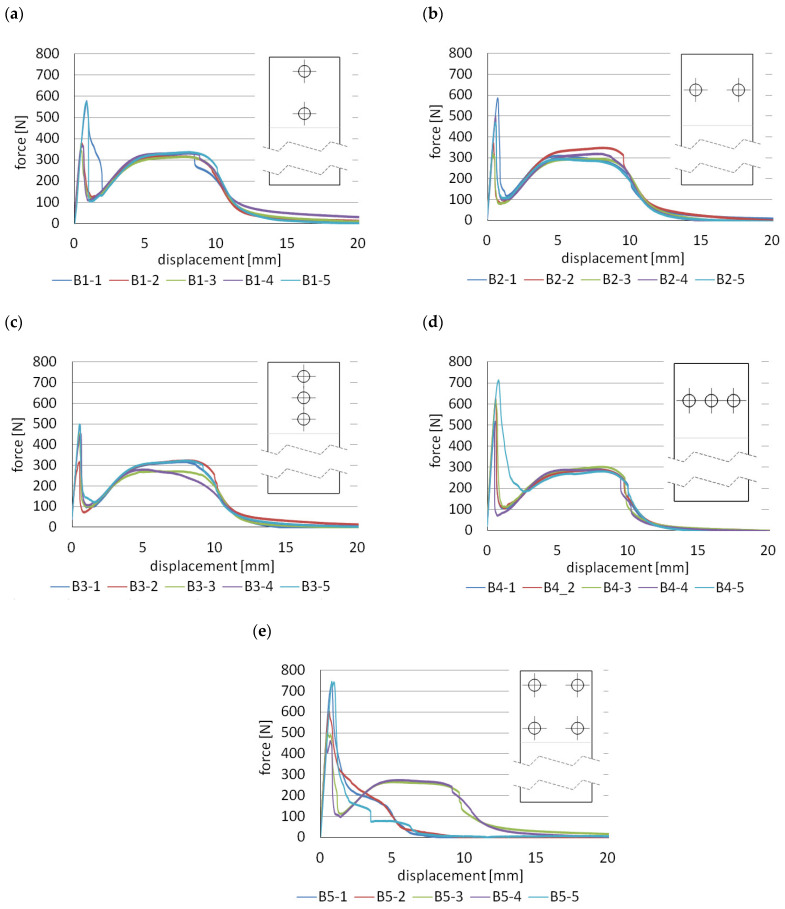
Force-displacement diagrams for “B” models: (**a**) B1 models; (**b**) B2 models; (**c**) B3 models; (**d**) B4 models; (**e**) B5 models.

Type B specimens, for which the force-displacement diagrams are shown in Figure 6, were made and tested to determine the effect of the number of mechanical joints on their mechanical response. In this case, the polymeric tenon was 5 mm in diameter for each specimen, having two, three and four tenons. Models B1 (Figure 2a) and B2 (Figure 2b) had two tenons. In the first case, the line on which the tenons were placed, coincided with the axis of the specimen and thus the line of load action. In the second case, the line connecting the two pivots was perpendicular to the line of load action and was located at the midpoint of the lap length (Figure 2b). Failure of single-lap joints often starts at the free edge of the joint between two materials, where there is a stress singularity leading to cracks developing exponentially [39]. Hence, mechanical fasteners are most advantageously placed near the end of the lap region [18]. It is also common to introduce an additional pre-stressing force from the mechanical fastener [40]. 

Table 3 collects the force values F_1_–F_3_ and the corresponding stress values σ_1_–σ_3_ in the 3D printed lap outside the joint zone, assuming that it is only in tension.

In the tests conducted, similar values of F_1_ forces were obtained for B1 and B2 specimens; however, a higher value of F_3_ force and energy in relation to B2 specimens favours placement along the line of force action.

The next batches of samples to be analysed are groups of specimens B3 and B4. These are analogous to series B1 and B2, the only difference being the introduction of an additional third tenon. 

In this case, it is more advantageous to place the row of mechanical joints, perpendicularly to the loading direction. This results in an increase of approximately 19.5% in the F_1_ force level, compared to specimen B3. Both F_2_ and F_3_ force and energy levels remain the same for both series types.

A different model is that of the B5 group of specimens in which one additional mechanical joint was introduced. These were also repositioned so that they were located at the corners of the overlap. There is an increase in F_1_ force of about 77% over the reference model. Although specimen B5 had the smallest adhesive tape area, the energy required to failure is at a level similar to that for models B2, B3 and B4. To the disadvantage of model B5 is the fact that three specimens in this series failed in an unusual way, consisting in the absence of the working stage of the double-sided adhesive tape. Once the maximum F_1_ force is reached, there is a decrease in its value with varying speed. Initially, the decrease is rapid—to about half of the F_1_ value—but then the graphs become smoother. From this, it can be concluded that the greater the number of mechanical joints, the greater the influence of defects, both in the making of the holes in the tape and in the influence of clearances in the mechanical joints. In order to prevent clearances between tenon and mortise, the authors in [15] proposed an original method by heating a metal lap and forming the heated tenon under pressure. This type of technology can eliminate the clearance; however, its application, in many cases, may be limited because the heating process itself takes a certain amount of time and must be strictly controlled. In order to obtain proper pressure and the plasticisation of the tenon in the groove, the joint must be supported on the other side. Manufacturing this type of specimen and applying it in engineering may be difficult to achieve, especially due to lack of available space.

**Figure A10:**
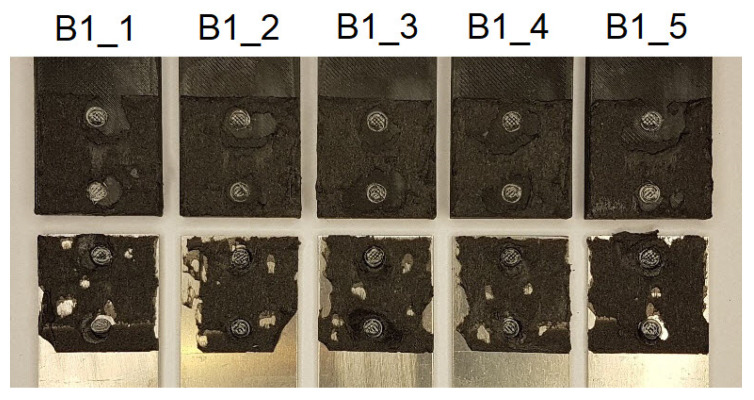
Samples B1 after the tensile test.

**Figure A11:**
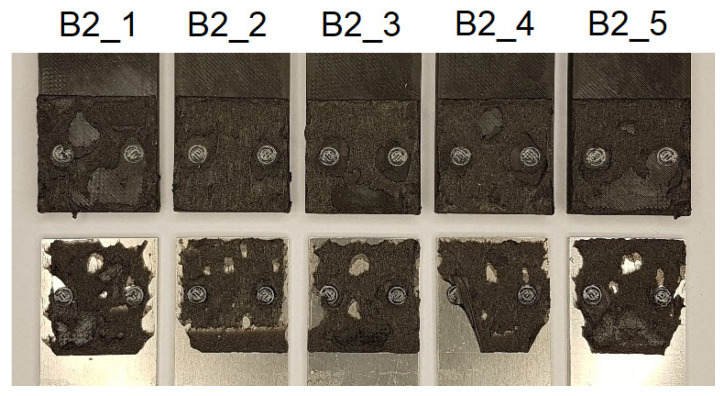
Samples B2 after the tensile test.

**Figure A12:**
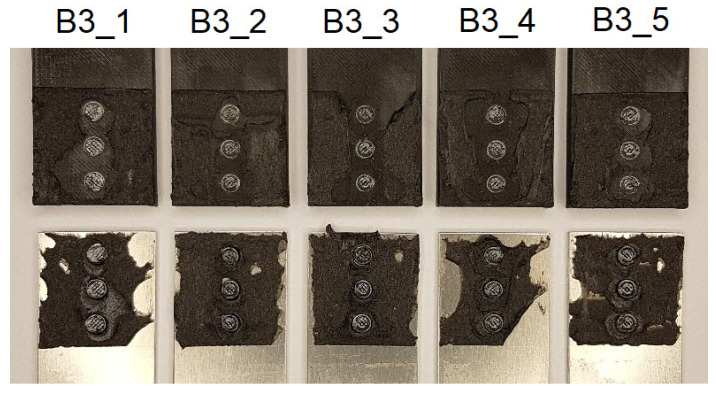
Samples B3 after the tensile test.

**Figure A13:**
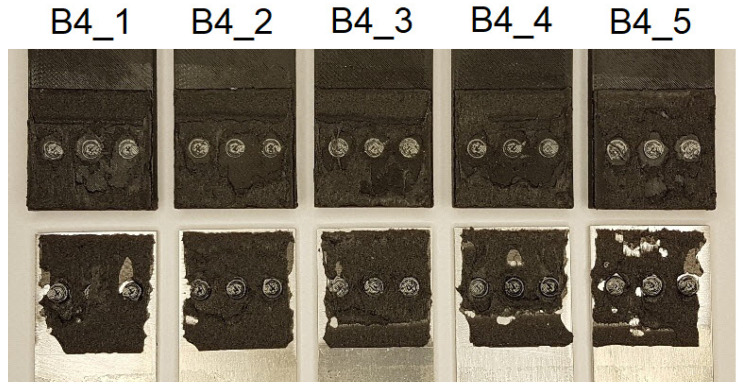
Samples B4 after the tensile test.

**Figure A14:**
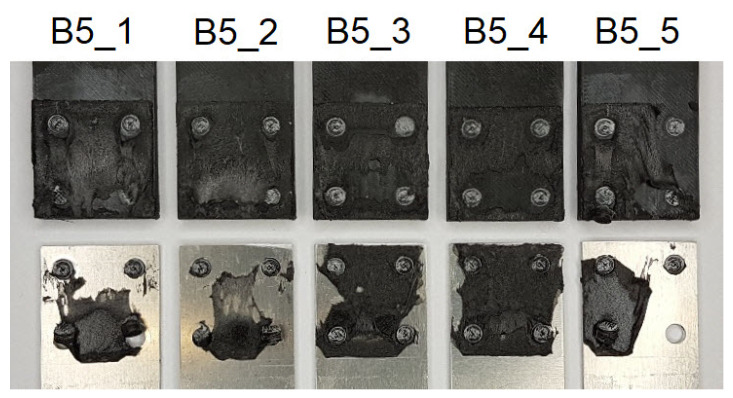
Samples B5 after the tensile test.

In conclusion, the most advantageous solution from the group of B models is model B1. In this case, both an increase in the value of F_1_ force by about 14% in comparison with the reference model “R” and an increase in the energy required to failure of the joint by 10%, are obtained. In addition, the joint shows significant stiffness at the stage of reaching the F_1_ value, while operation of the double-sided adhesive tape provides high safety, since its degradation consumes the most energy. The samples after the test are shown in Figure A10, Figure A11, Figure A12, Figure A13 and Figure A14. The cohesive nature of the failure that is characteristic of double-sided VHB tape is most disturbed for samples B5 (Figure A14). In this case, the cohesive failure covers about 46% of the total surface area, the adhesive failure between the tape and the aluminium surface is as high as 41%, and the adhesive failure between the tape and the 3D printed polymer is 13%, considering all the samples in the batch. 

### 3.4. Mechanical Response of Hybrid Joints Characterised by Multi-Stage Operation—C Group

The last type of “C” models is the most original and advanced. The idea of this type of connection is to achieve multi-stage operation and specific characteristics, through the use of mechanical joints that take part in load transfer in a controlled manner. This is realised by an appropriately sized clearance in one of the laps, by making an elongated hole. In this type of model, there can be different solutions:

(a) all mechanical joints have the same diameter and the holes with which they engage are equally elongated (e.g., C1 Figure 3a),
(b) both tenons are seated in holes of the same diameter and tenons engage with elongated holes of the same length (e.g., C2 Figure 3b, C3 Figure 3c),
(c) there are tenons embedded in holes of the same diameter and tenons engaging with elongated holes of different lengths (e.g., C4 Figure 3d, C5 Figure 3e).

**Figure 7:**
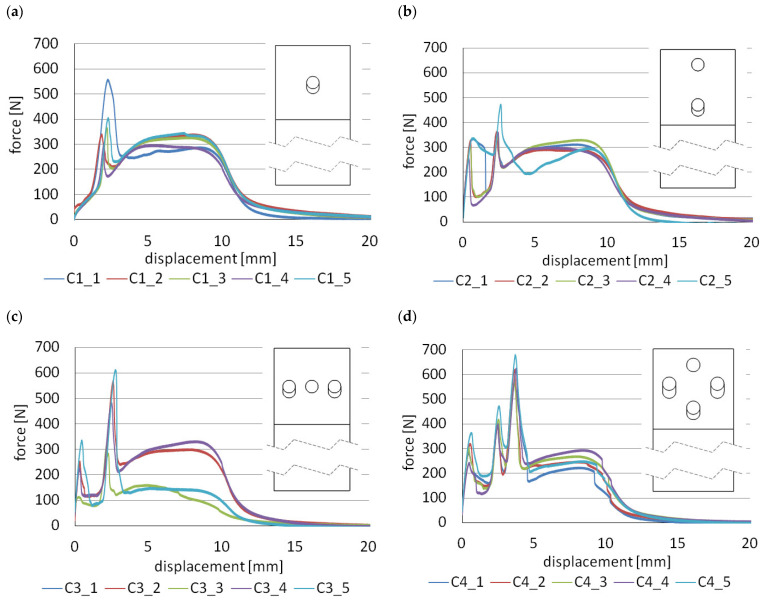

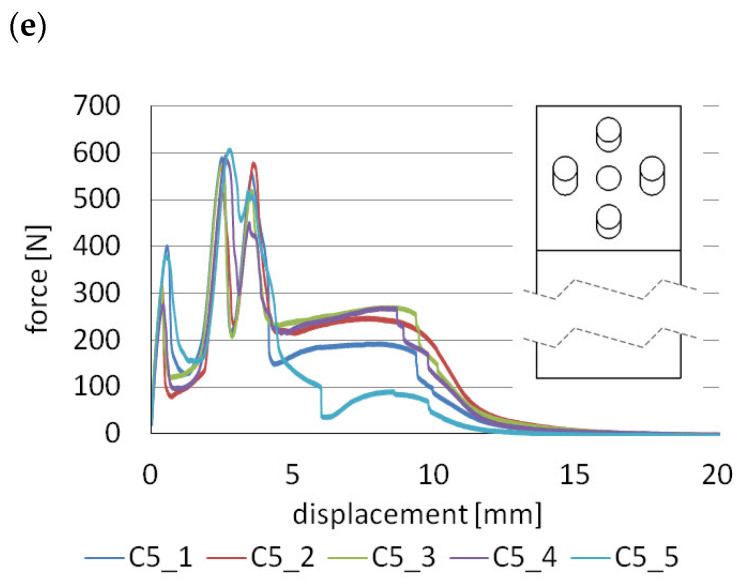
Force–displacement diagrams for “C” models: (**a**) C1 models (Figure 3a); (**b**) C2 models (Figure 3b); (**c**) C3 models (Figure 3c); (**d**) C4 models (Figure 3d); (**e**) C5 models (Figure 3e).

**Figure 8:**
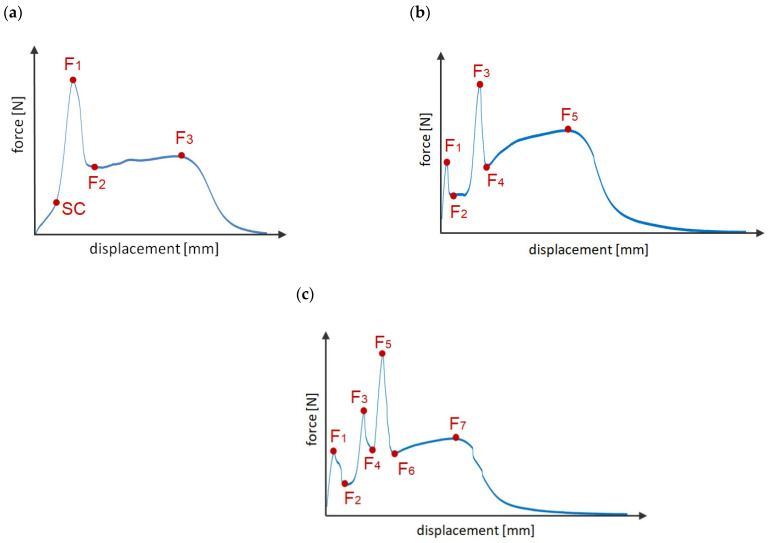
Schematics of connections: (**a**) C1; (**b**) C2; C3; (**c**) C4; C5.

**Figure A15:**
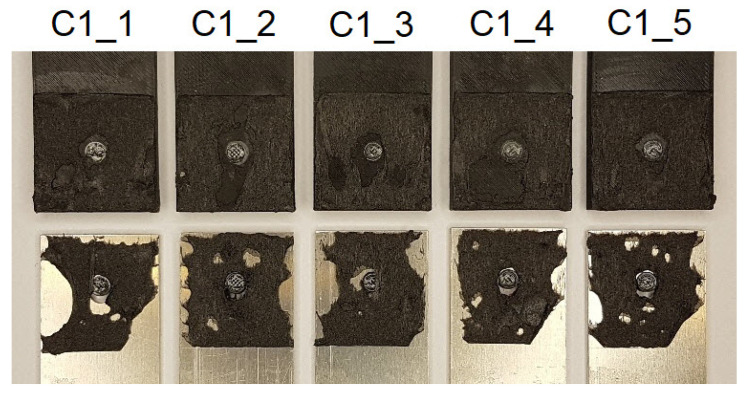
Samples C1 after the tensile test.

**Figure A16:**
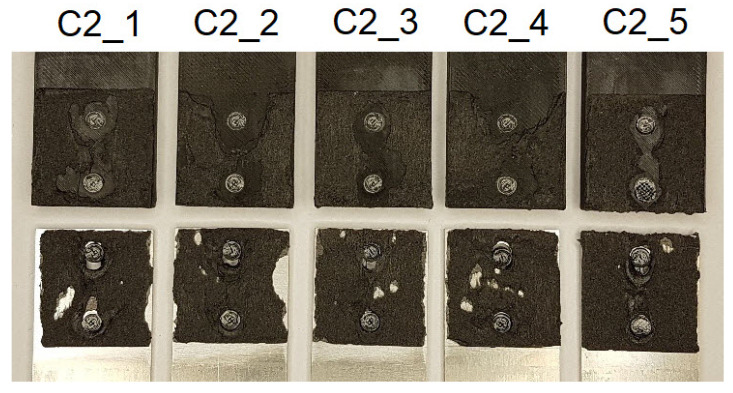
Samples C2 after the tensile test.

**Figure A17:**
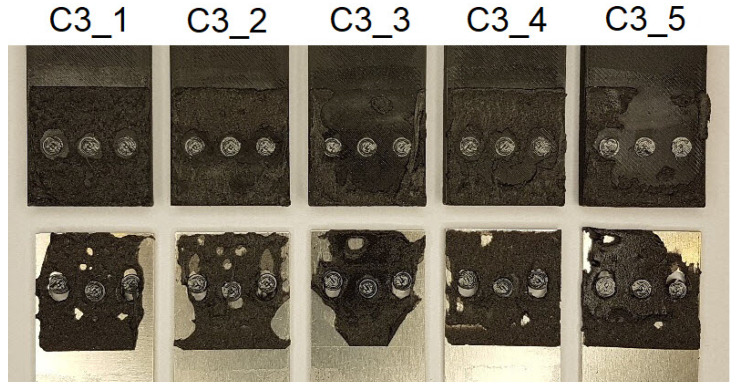
Samples C3 after the tensile test.

**Figure A18:**
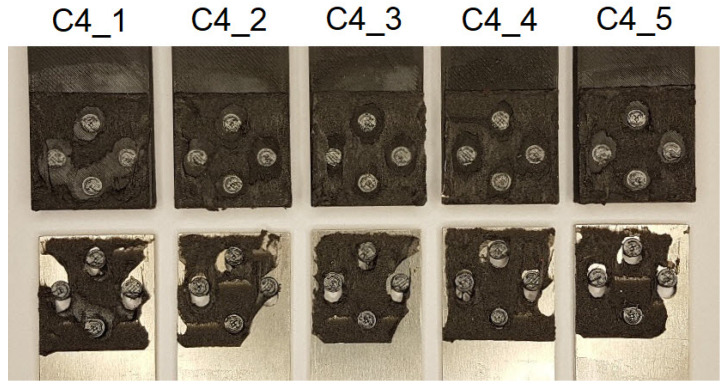
Samples C4 after the tensile test.

**Figure A19:**
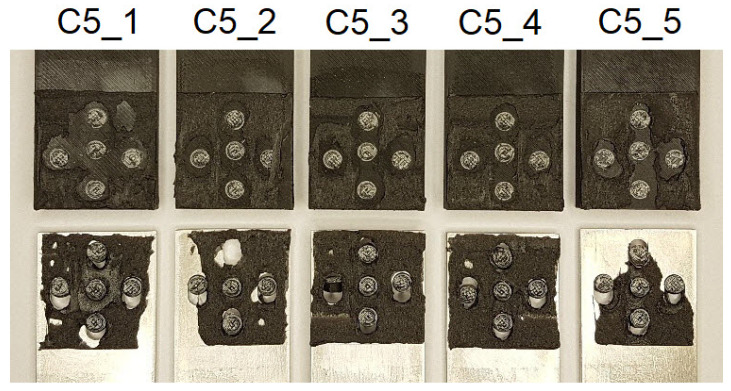
Samples C5 after the tensile test.

Figure 7 shows the force–displacement diagrams obtained from the laboratory tests, while Figure 8 shows the three different characteristics that correspond to them. The samples after the test are shown in Figure A14, Figure A15, Figure A16, Figure A17, Figure A18 and Figure A19. For example, for specimen C1 (Figure A15), the cohesive failure covers 71% of the lap area, the adhesive failure between the tape and the aluminum surface is 19%, and the adhesive failure between the tape and the 3D printed polymer is 10%, considering all specimens in the series.

For specimen C1, the scheme of operation is similar to models A and B, whose characteristics are shown in Figure 5f. In specimen C1, there is an elongated hole, and it is only after some time, with the joint beginning to stretch, that the tenon engages with the hole. Therefore, there is a change in stiffness as indicated by the SC (stiffness change) point in Figure 8a. By changing the length of the hole, it is possible to design which displacement (load) the joint starts to resist more. Further operation takes place as for models A and B and three levels of forces F_1_, F_2_, and F_3_ can be distinguished.

The second group of models are C2 and C3. Their operating characteristics are shown in Figure 8b. 

Already, from the beginning of the load, with these models, one of the tenons engages with the hole. The joint therefore has a much higher stiffness than if only the double-sided adhesive tape was working i.e., model “R”. In the next stage, when force F_1_ is reached, the tenon is cut due to shear and the force drops to F_2_, the load being carried by the adhesive tape. In the third stage, other mechanical joints engage with the holes and the force increases again to F_3_. The fourth and final stage occurs after all tenons have been sheared and the load is again taken up by the adhesive tape. Of course, force levels F_1_ and F_2_ can differ, depending on the diameter value and the number of tenons for each stage.

The last group of joints C4 and C5 (Figure 8c) is the most advanced because there are as many as six stages of operation—three stages of mechanical joint operation and three stages of adhesive tape operation. To achieve this effect, one of the laps must have both holes with diameters corresponding to the mating tenons and two types of enlarged holes. By properly selecting the diameters of the tenons and the length of the enlarged holes, it is possible to obtain different force ratios F_1_, F_3_, F_5_ and F_7_. The disadvantage of this type of solution is that more effort is required to drill the holes and high precision must be exercised.

Table 4 collects the force values F_1_–F_7_ and the corresponding stress values σ_1_–σ_7_ in the 3D printed lap outside the joint zone, assuming that it is only in tension. Analysis and discussion of the results for the C series will begin with a comparison of the A1 and C1 models. Both types of connections have one tenon with the same diameter of 5 mm. According to Table 2 and Table 4, the F_1_ forces are 246 N and 340 N, respectively. Thus, the application of additional clearance in the hole allows an increase in the F_1_ force of about 38%. This large increase can be explained by the fact that, in the case of model A1, the double-sided adhesive tape is completely relieved when it is loaded. In the case of model C1, there is already considerable deformation in the tape and the mechanical fastener engages smoothly with the whole joint. However, there is a question as to what the value of the F_1_ force would be if the mechanical fastener were to come into contact with the hole, at the stage where the double-sided adhesive tape reached its maximum force value, i.e., in the displacement range of about 4–9 mm. This type of model will be the subject of on-going laboratory tests. Note, however, that, for model C1, there is a change in stiffness (point SC of Figure 8a). 

In the four-stage C3 model, the predicted effect of giving a larger F_3_ force was obtained compared to the C2 model. This effect was achieved by having two tenons come into contact with a delay rather than one, as is the case in model C2. The percentage difference in this case is approximately 21%. 

The common feature of C4 and C5 models is that, in the initial stage, one tenon is working; therefore, F_1_ forces are at a similar level. The second and third stages of the mechanical connections are already different, due to the different number of tenons, coming in contact with the holes; hence, forces F_3_ and F_5_ are different. For the C5 models, the lowest value of force F_7_ is also obtained and is 36% lower compared to the reference model “R”. The explanation for this decrease is related to the making of as many as five holes in the adhesive tape, which results in a weakening of the tape. The use of one or two tenons in the lap does not reduce the energy required for them to fail. For models C1 and C2, the same values were obtained as for the reference model “R”. However, the energy reduction is very negatively affected by placing three tenons in one row, perpendicular to the direction of the load action. In this case, the decrease of this parameter is as much as 23%. Similarly, for model B4, the lowest energy value in the entire “B” series was also obtained.

### 3.5. Scatter Analysis of the Results

**Figure 9:**
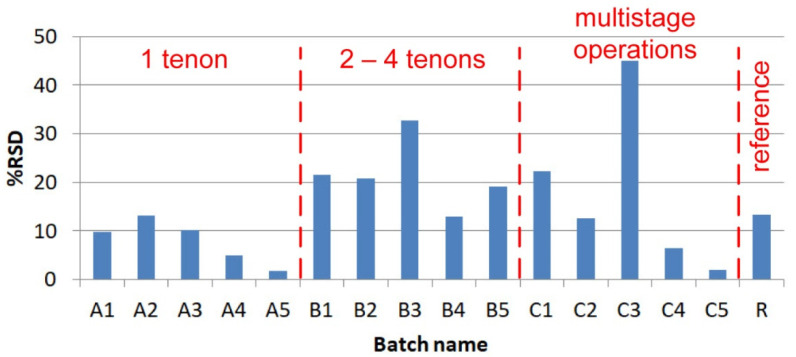
Relative standard deviation.

Figure 9 shows the relative standard deviation for all series for maximum force. The “R” reference samples achieved a value of 13.3%. This is not a large value considering that operations such as cutting the double-sided tape and joining the laps were performed manually, without the use of special positioning devices. Therefore, one cannot have any objections to the “A” series. In this case, the maximum value was obtained for model A2 and was 13.2%. Samples A5 have a very low % RSD parameter because, in this case, there was a breakage of the polymer laps. The “B” group is dominated by values of 20%; only samples B3 obtained a % RSD level of almost 33%. The increase in value of this parameter is associated with a higher degree in the complexity of the geometry, making more holes, which must be fitted properly. Interesting results were obtained for the “C” group; on the one hand, there is a very low repeatability of results in relation to the maximum force for C3 samples (45% RSD). On the other hand, there is also a very low value of 2% for specimen C5, which can be explained by the fact that the fasteners are uniformly distributed in the lap and come into contact with the holes in a smooth manner.

The nature of the joint failure using VHB tape may also influence the scatter in results. Theoretically, only cohesive failure should occur in the tape volume, but, regardless of the type of specimen, there is additionally adhesive failure between the tape and the surface of the aluminium adherend and between the tape and the surface of the 3D printed polymer. The largest share of cohesive failure (about 74.6%) occurs in the reference samples. After the insertion of tenons in the B and C series samples, the percentage of cohesive failure decreases. The reason may be the fragments of tenons after their failure, which, moving together with the lap, cause tearing of the tape and change the nature of its failure to adhesive. 

The results of the laboratory tests obtained can be a valuable source of data for calibrating numerical models, which is necessary because, as shown in the current paper, the number of combinations of the use of mechanical joints can be very large.

## 4. Conclusions

This paper presents three groups of original and innovative single-lap joints that are made using double-sided adhesive tape and mechanical joints by tenons. The manner in which their manufacture is implemented is described in detail. The study of lap joints was carried out on a total of 80 samples in which one of the laps was 3D printed, Z-ABS material and the other was made of aluminium. Joining of 3D printouts is a new issue and has been put into practice in many ways. However, no connections as advanced as those presented in this paper have been encountered. As the results of the tests illustrate, the following conclusions can be formulated:

- the introduction of mechanical joints, depending on the number and diameter of tenons, does not cause too high an increase in the energy required to cause the joints to fail, in comparison to the reference model R (maximum 10%). More often, there is a decrease of this parameter even by as much as 23%. This is because the polymeric tenon is much stiffer than the VHB tape, but it fails at small strains, hence the total energy is similar in each case,
- the introduction of mechanical connections significantly increases joint stiffness and maximum force. The largest increase was obtained for model A4 and was equal to 186%, compared to the reference model,
- the proportional increase of the tenon area, in the group A specimens, also results in a proportional increase in maximum force, with the operation of these joints being a two-stage process,
- increasing the tenon area, in the group A specimens, decreases the force transferred by the double-sided adhesive tape in the second stage, but all joints, regardless of tenon area, fail at the same displacement,
- the most advantageous solution from group B is model B1 which achieves both an increase in F_1_ force of about 14% in comparison to reference model R and above a 10% increase in the energy required to cause the joint to fail.
- specimens of the group C have the greatest potential for the arbitrary shaping of the characteristics of lap joints. The four- and six-stage joints, as designed, can significantly increase the safety of the structural elements,
- Four types of failure were observed during the study. The first is the cohesive failure that occurs in the volume of the VHB tape; the effect is that it remains on both surfaces of the laps. The second type consists of deformation and finally shearing of the polymeric tenons. The third type of failure, which occurs in almost every sample, is the loss of adhesion between the VHB tape and the laps. The last type of failure, which occurred in two A4 series specimens and all A5 series specimens, is cracking of the polymer laps at the tenon location.

## Figures and Tables

**Table 1:** Printing parameters and properties of ABS and 3D-printed specimens.

Printing Parameters	Values	ABS Parameters	Values
Raster angle (°)	±45	Density (g/cm^3^)	1.06
Raster width (mm)	0.4	Young modulus (GPa)	2.24
Print tolerance (%)	±0.2	Poisson ratio	0.38
Infill percentage (%)	100	Yield point (MPa)	20
Layer thickness (mm)	0.09	Tensile strength (MPa)	29.6
Bed temperature (°C)	80	Filament diameter (mm)	1.75
Nozzle diameter (mm)	0.4	Diameter tolerance (mm)	±0.05
Printing speed (mm/s)	30	Melting point (°C)	∼250
Nozzle temperature (°C)	275	Thermal conductivity (W/mK)	0.16

**Table 2:** Summary of average F_1_, F_2_, and F_3_ forces and the corresponding stresses σ_1_, σ_2_ and σ_3_ in the printed lap outside the joint zone as well as energies for Type A specimens.

	Model A1	Model A2	Model A3	Model A4	Model A5
F_1_ [N], (σ_1_ [MPa])	246 (1.64)	488 (3.25)	744 (4.96)	953 (6.35)	1094 (7.29)
F_2_ [N], (σ_2_ [MPa])	83 (0.55)	89 (0.59)	90 (0.6)	123 (0.82)	126 (0.84)
F_3_ [N], (σ_3_ [MPa])	342 (2.28)	329 (2.19)	277 (1.85)	219 (1.46)	207 (1.38)
energy [J]	3.1	3.2	2.8	3.1	3.3

**Table 3:** Summary of average F_1_, F_2_, and F_3_ forces and the corresponding stresses σ_1_, σ_2_, and σ_3_ in the printed lap outside the joint zone as well as energies for Type B specimens.

	Model B1	Model B2	Model B3	Model B4	Model B5
F_1_ [N], (σ_1_ [MPa])	380 (2.53)	395 (2.63)	451 (3.01)	539 (3.59)	591 (3.94)
F_2_ [N], (σ_2_ [MPa])	147 (0.98)	101(0.67)	137 (0.91)	144 (0.96)	104 (0.69)
F_3_ [N], (σ_3_ [MPa])	325 (2.16)	305 (2.03)	287 (1.91)	288 (1.92)	270 (1.8)
energy [J]	3.3	2.8	2.8	2.7	2.8

**Table 4:** Summary of average F_1_–F_7_ forces and the corresponding stresses σ_1_–σ_7_ in the printed lap outside the joint zone as well as energies for Type C specimens.

	Model C1	Model C2	Model C3	Model C4	Model C5
F_1_ [N], (σ_1_ [MPa])	340 (2.32)	285 (1.90)	219 (1.46)	291 (1.94)	310 (2.07)
F_2_ [N], (σ_2_ [MPa])	233 (1.55)	151 (1.01)	98 (0.65)	156 (1.04)	124 (0.83)
F_3_ [N], (σ_3_ [MPa])	-	347 (2.31)	420 (2.8)	411 (0.27)	552 (3.68)
F_4_ [N], (σ_4_ [MPa])	-	241 (1.61)	184 (1.23)	246 (1.64)	329 (2.19)
F_5_ [N], (σ_5_ [MPa])	-	-	-	598 (3.99)	514 (3.43)
F_6_ [N], (σ_6_ [MPa])	-	-	-	221 (1.47)	197 (1.31)
F_7_ [N], (σ_7_ [MPa])	313 (2.09)	300 (2.00)	226 (1.51)	251 (1.67)	212 (1.41)
energy [J]	3	3	2.3	2.8	2.5

## Data Availability

The data presented in this study are available on request from the corresponding author.
